# Primary Intracranial Adenoid Cystic Carcinoma: A Case Report

**DOI:** 10.31729/jnma.8890

**Published:** 2025-02-28

**Authors:** Pralisha Maharjan, Gopi Aryal, Reena Rana

**Affiliations:** 1 Department of Laboratory Medicine and Pathology, Nepal Mediciti Hospital, Lalitpur, Nepal

**Keywords:** *adenoid cystic carcinoma*, *craniotomy*, *intracranial*

## Abstract

Adenoid cystic carcinoma (ACC) is a rare slow-growing but aggressive malignant tumor arising from the epithelial cells of mucous-secreting glands. Primary intracranial ACC is one of the rarest entity. We report a case of a 61 years old male presenting with difficulty in swallowing, slurring of speech, generalized body weakness. Patient had residual right cerebellopontine angle (CPA) mass causing midline shift and fourth ventricular obstruction on MRI. Patient underwent right retrosigmoid craniotomy with excision of CPA mass. Histopathological examination confirmed the case as primary intracranial ACC.

## INTRODUCTION

Adenoid cystic carcinoma (ACC) is a rare slow-growing but aggressive malignant tumor with potential of perineural invasion. It accounts for less than 1% of all head and neck malignancies and approximately 10% of all salivary gland neoplasms. ^1^ Primary intracranial ACC is one of the rarest entity. ACC has three main histological patterns : solid, tubular, and cribriform. Only eight cases of primary intracranial ACC have been reported till date (1990-2022). ^2,3^ Here we report a case of 61 years male diagnosed with primary intracranial ACC.

## CASE

A 61 years man presented with dysphagia, slurring of speech, generalized body weakness for three months. On clinical examination, he was conscious and oriented, his vitals were stable and on other systemic examinations no abnormalities were detected . On neurological examination, higher motor function was intact but patient had right sided lower motor neuron facial palsy of grade III and right sided sensorineural hearing loss, cerebellar signs were positive on right side and had gait ataxia, gag reflex was absent and there was no motor and sensory deficits. Hematological and biochemical parameters were within normal limits. The patient had significant past history of right CPA mass for which he was operated at other medical center and histological report was suggestive of neuroenteric cyst. Repeated Magnetic Resonance Imaging (MRI) showed tumor infiltrating the nerve sheath of cranial nerves 9th , 10th and 12th on right side extending into exocranial aspect and inferiorly along pterygoid muscle, lateral wall of nasopharynx and right medial temporal region for which intervention was not done.

**Figure 1:**
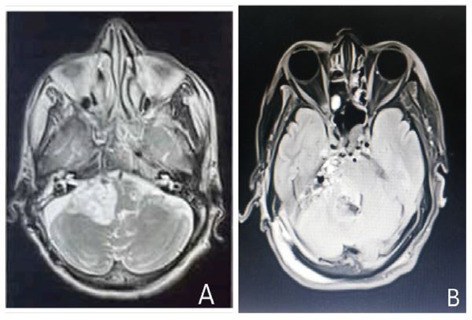
A. Preoperative MRI brain revealing a hyperintense lesion on T2-weighted image on axial plane measuring 4.2 cm × 4.0cm × 1.4cm. B. Postoperative MRI brain revealing heterogenous collection at tumor resection bed in right lateral aspect of posterior fossa where central component of the collection displays mixed signal.

The MRI done on 15th April, 2024 revealed right CPA mass causing midline shift and fourth ventricular obstruction (Figure 1). He underwent right retrosigmoid craniotomy with excision of CPA mass on 23th April, 2024. Peroperatively, the lesion was greyish white to greyish brown and firm.

**Figure 2:**
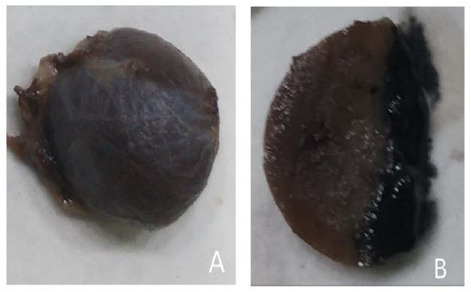
A. Gross image appeared greyish white to greyish brown nodular tissue. B. Cut section showed solid to cystic area.

On gross examination, the specimen appeared greyish brown with largest nodular tissue measuring 1.5cm×1.2cm (Figure 2A). Cut sections showed solid to cystic area. (Figure 2B). Remaining fragmented tissue altogether measures 2.5×2.2cm. Entire tissue submitted.

**Figure 3:**
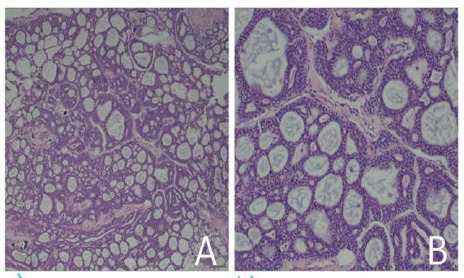
A. Tumor tissue predominantly composed of cribriform growth pattern with nest of tumor cells with discrete, rounded punched out gland like spaces filled with eosinophilic to basophilic material (Hematoxylin and Eosin stain x10). B. Area of tubular pattern with multiple ducts and tubules like structure are lined by small uniform cuboidal epithelium (Hematoxylin and Eosin stain x40).

Microscopic examination revealed tumor tissue predominantly composed of cribriform growth pattern with nest of tumor cells with discrete, rounded punched out gland like spaces filled with eosinophilic to basophilic material (Figure 3A). Area of tubular pattern with multiple ducts and tubules like structure are lined by small uniform cuboidal epithelium (Figure 3B). A focus of solid sheets of tumor cells (<30% solid component) noted. Infiltrating tumor nests are identified within fibrous stroma. Perineural invasion is evident. Focal areas of reactive gliosis with dilated and congested blood vessels are noted.

**Figure 4:**
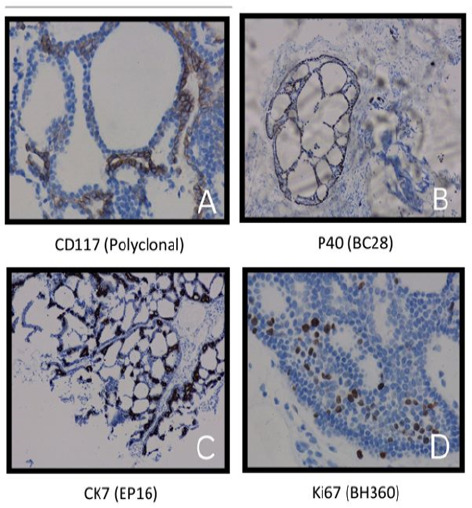
A. The tumor cells expressed CD117 positivity in luminal layer. B and C. The tumor cells expressed P40 and CK7 in myoepithelial layer. D. Ki-67 was nearly 12%

The tumor cells expressed CD117 positivity in luminal layer and P40 and CK7 in myoepithelial layer but was negative for S100 and Ki-67 was nearly 12% (Figure 4). With all these histological features and immunohistochemical reports, the case was finally diagnosed as intracranial ACC.

To look for the primary site of the lesion, further evaluation like chest X-ray, ultrasonography of abdomen, and whole-body MRI was performed but the primary site remained unknown so we concluded the tumor to be intracranial in origin and diagnosed it as primary intracranial ACC. Patient was managed conservatively with intravenous antibiotics and all other supportive measures. However, the patient didn't receive postoperative radiotherapy and he passed away on 18th post operative day (POD).

## DISCUSSION

ACC is a rare slow-growing but aggressive malignant tumor. More than half of ACC arises from the minor salivary glands but it can also affect the major salivary glands, paranasal sinuses, larynx, and trachea and hard palate is most commonly affected intraoral site.^4^ Though direct extension of the tumor or invasion along cranial nerves is common but primary intracranial ACC is extremely rare. Only eight cases of primary intracranial ACC have been reported till date (1990-2022) ^2,3^ where the youngest case was 34 years and the eldest was 71 years, clinical features varied from headache, epistaxis to hemianopia and left facial region numbness and most common location was cavernous sinus. Cases underwent operation, radiosurgery and radiotherapy. Among them 3 cases died after management while 2 cases were alive with disease and 3 were alive without disease.

In our case, the mass was seen in right CPA causing midline shift and fourth ventricular obstruction. Literatures have shown that the tumors arising close to the skull base may cause ocular dysmotility, and cranial nerve (CN) palsies involving IX, X, XI, and XII.^5,6^ It is thought to arise from existing bucconasal cell rests.^7^ Among the primary intracranial ACCs reported in the literature, Gasserian ganglion, middle cranial fossa, frontal lobe, cavernous sinus and posterior fossa comprise majority of locations.^8^ Similar to findings of these literatures, our case also had multiple nerve sheath tumors (right IX CN, X CN, XII CN), extending into exocranial aspect and inferiorly along pterygoid muscle, lateral wall of nasopharynx and right medial temporal region as seen on MRI.

Age of the patient, tumour site, clinical stage, histological type with or without nerve involvement are the important factors influencing prognosis however the survival at 5,10, and 20 years around 68%, 52% and 28% respectively after decompression surgery and postoperative radiotherapy.^9^ The need for early diagnosis and radical tumor resection, if possible , coupled with postoperative radiation should be emphasized so that local disease control and better long-term survival will be achieved.^10^ However, the patient didn't receive postoperative radiotherapy and he passed away on 18^th^ POD .
